# Deep Brain Stimulation in Early-Stage Parkinson’s Disease: Patient Experience after 11 Years

**DOI:** 10.3390/brainsci12060766

**Published:** 2022-06-11

**Authors:** Kaitlyn Stoehr, Kian Pazira, Kemberlee Bonnet, David Schlundt, David Charles, Mallory Hacker

**Affiliations:** 1 Department of Neurology, Vanderbilt University Medical Center, Nashville, TN 37232, USA; kaitlyn.a.stoehr@vanderbilt.edu (K.S.); kian.pazira@vanderbilt.edu (K.P.); david.charles@vumc.org (D.C.); 2 Department of Psychology, Vanderbilt University, Nashville, TN 37235, USA; kemberlee.bonnet@vanderbilt.edu (K.B.); david.schlundt@vanderbilt.edu (D.S.)

**Keywords:** Parkinson’s disease, deep brain stimulation, qualitative research, patient experience, clinical trials

## Abstract

The deep brain stimulation (DBS) in early-stage Parkinson’s disease (PD) pilot trial began more than a decade ago and remains the only investigation of DBS in mildly symptomatic patients. Patients completed therapeutic washouts biannually for two years, outpatient assessments through five years, and a longitudinal washout assessment after 11 years. Here, the patient experience of participating in the early DBS pilot trial is described. Semi-structured interviews were audio-recorded and transcribed. Transcripts were coded, analyzed using an iterative inductive-deductive approach, and used to develop a conceptual framework. Ten participants (n = 6 early optimal drug therapy (ODT), n = 4 early DBS + ODT) were interviewed. Motivations for participation included benefit to future PD patients and potential personal benefit, while hesitations included risk of surgical complications. While early ODT patients who received standard-of-care DBS described significant changes in their functional capacities after surgery, early DBS patients described a maintenance of quality of life that made PD less impactful over an extended period. Patients expressed high satisfaction with trial participation and early DBS. This study suggests that the PD experience with early DBS may notably differ from standard-of-care DBS. The FDA has approved the conduct of a pivotal clinical trial evaluating DBS in early-stage PD (IDEG050016).

## 1. Introduction

Deep brain stimulation (DBS) is an adjunctive treatment currently offered to patients with mid-to-advanced stage Parkinson’s disease (PD) that provides significant improvement in motor symptoms, reduces medication burden, and enhances quality of life [1,2,3,4,5,6]. Qualitative research has provided insight into the patient experience of living with DBS. Advanced-stage patients describe the period before receiving DBS as a time in which symptom progression has led to a loss of control and unpredictability, causing them to consider DBS, often as a last-resort therapy [7,8]. After receiving DBS, patients often describe feelings of liberation from pain and off episodes and a renewed freedom to perform normal activities [7,9]. This is typically followed by a period of transition, in which patients experience new opportunities to live more fully but also must adapt to new challenges. Our current understanding of the PD patient experience with DBS is appropriately based on the therapy’s present indication: after dyskinesia or motor complications have developed and medications no longer adequately control symptoms (i.e., mid-to-advanced-stage disease).

The robust benefits of DBS in later stages of PD [1,2,3,4,5,6], along with animal studies suggesting that early DBS may have neuroprotective effects [10,11,12,13], support investigations into DBS applied in very early-stage PD. The first clinical trial of early DBS demonstrated initial safety and tolerability [14] (IDEG050016, NCT00282152). All 29 subjects who completed the pilot trial enrolled in an observational follow-up study where they were evaluated annually through five years [15]. Results from the five-year follow-up study provided Class II evidence that DBS implanted in early-stage PD decreases the risk of disease progression and polypharmacy compared to optimal medical therapy alone [15]. Recently, 12 patients from the early DBS trial participated in a longitudinal follow-up study to evaluate 11-year changes in motor and cognitive outcomes.

In addition to safety and efficacy, which will be evaluated in a future pivotal clinical trial (IDE#G050016), patient experience is a key consideration in determining the viability of early DBS therapy. Unlike patients who receive DBS as a last-resort treatment per standard care, patients from the early-stage PD pilot trial offer the unique perspective of receiving the therapy while only mildly symptomatic. Therefore, to understand the patient perspective of PD progression after receiving early DBS, we interviewed 10 patients 11 years after enrolling in the DBS in early-stage PD pilot trial. Additionally, this cohort provides insight into the patient experience of a novel trial design that involves repeated week-long therapeutic washouts designed to capture PD symptoms in the untreated state. This study describes the long-term patient experience of participating in the only trial of DBS in early-stage PD.

## 2. Materials and Methods

### 2.1. Design

#### 2.1.1. DBS in Early PD Pilot Trial

**Figure 1:**
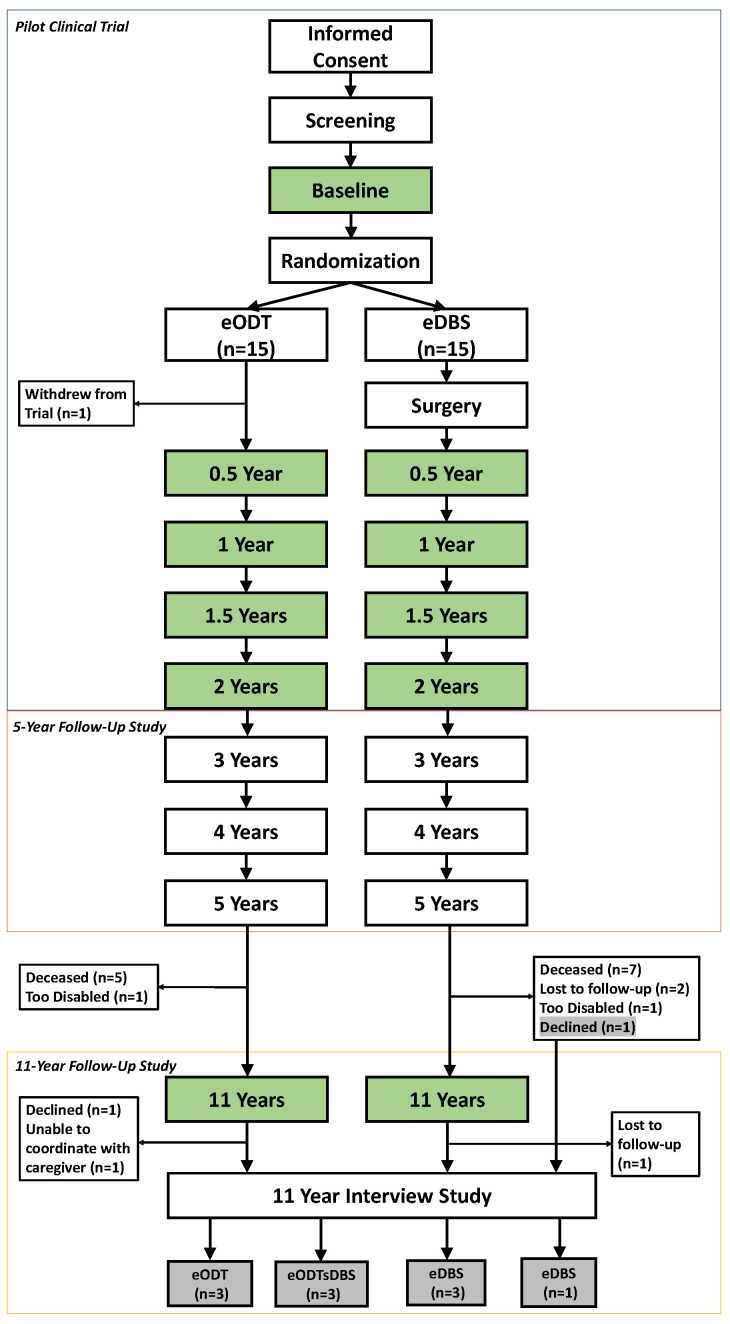
**DBS in early PD pilot trial participants in this 11-year interview study.** Thirty participants enrolled in the pilot clinical trial and were randomized 1:1 to receive bilateral subthalamic nucleus deep brain stimulation (eDBS) plus optimal drug therapy (ODT) or ODT alone (eODT) and followed for two years. Week-long therapeutic washouts (green boxes) were conducted every six months during the two-year trial. All participants who completed the trial enrolled in a five-year follow-up study. Twelve patients completed an 11-year study visit, which included the week-long therapeutic washout (green boxes). Ten patients from the DBS in early-stage PD pilot clinical trial participated in this 11-year interview study. eODTsDBS = randomized to early ODT and later received DBS as standard of care after the trial completed.

The DBS in early-stage PD pilot was a prospective, randomized, controlled, single blind clinical trial (NCT00282152; IDEG050016; IRB0470797). The trial enrolled patients 50–75 years old with very early-stage PD (Hoehn and Yahr II off medication; medication duration 0.5–4 years; without history of dyskinesia or other motor fluctuations) [16]. An expanded informed consent process was used to help potential participants understand the goals and the risks of the trial [17]. After providing written informed consent, 30 patients who passed screening completed a week-long therapeutic washout in the Clinical Research Center (CRC) at baseline and were then randomized 1:1 to early DBS + ODT (eDBS) or early optimal drug therapy alone (eODT). The week-long washout was repeated biannually during the two-year trial (Figure 1), where patients completed clinical assessments including daily Unified Parkinson’s Disease Rating Scale Motor Evaluation (UPDRS-III) ratings both on and off PD therapy (off PD medications at baseline/before randomization; off PD medications and off STN-DBS stimulation, if applicable, at 6, 12, 18, and 24 months). Participants were informed that they may discontinue the washout at any time. Additional details describing the week-long washout are previously reported [14]. All patients who completed the two-year trial (n = 29) elected to participate in an observational follow-up study where they completed outpatient assessments at years 3, 4, and 5 [15]. Twelve patients (n = 4 eDBS, n = 8 eODT) participated in an 11-year follow-up visit, where the washout was repeated alongside prior study assessments and with the addition of exploratory metabolic (FDG) PET scans on day 4 (IRB180766). Three eODT participants in the 11-year study elected to receive DBS as standard care after the two-year trial completed (eODTsDBS).

#### 2.1.2. Interview Study

All patients previously enrolled in the early DBS pilot trial (NCT00282152; IDEG050016; IRB040797) who completed the 5-year outpatient follow-up study were approached by phone to participate in this sub-study (IRB191728). This interview study includes 9 out of the 12 patients who completed the 11-year follow-up study and 1 patient who declined to participate in the 11-year follow-up study but agreed to be interviewed. Reasons for non-enrollment included inability to coordinate with patient’s caregiver (n = 1), decision not to participate (n = 1), and loss to follow-up (n = 1; Figure 1). All patients provided informed consent.

### 2.2. Data Collection

An interview guide was developed with input from Vanderbilt University Qualitative Research Core (VU-QRC) personnel (D.S., K.B.). Due to the unique nature of the patient group, the interview guide was not pilot tested. Interview questions covered topics of the impact of the disease on patients’ lifestyle over time, patients’ expectations and satisfaction with treatment, patients’ judgements of practicability of the early DBS treatment, and patients’ washout experiences.

Telephone interviews were conducted by a VU-QRC coordinator (K.B., MA in Social Psychology, female, 10 years qualitative research experience) within 1.5 years of completing the 11-year follow-up study visit. The interviewer had no prior relationship with study participants. Mean interview duration was 40 min (23 min–64 min), and three participant interviews had caregivers present. Audio-recorded interviews were stored on an encrypted drive on a secured server until transcribed by the IRB-approved service rev.com.

The Parkinson’s Disease Quality of Life Questionnaire 39 (PDQ-39) was administered at baseline for all participants and at 11 years for the 9 out of 10 participants who completed the 11-year study visit.

### 2.3. Data Analysis

Qualitative data coding and analysis was managed by the VU-QRC, led by a PhD-level psychologist (D.S.). A hierarchical coding system was developed and refined using the interview guide and a preliminary review of the transcripts. Top-level categories in the coding system: (1) symptoms, (2) quality of life, (3) emotions, (4) washout, (5) DBS experiences, (6) change over time, (7) study experience, and (8) PD experience. Each category had 4–16 subcategories, some of which included three additional levels of hierarchical division. Definitions and rules were written for the use of coding categories.

Two trained qualitative coders (K.S., K.B.) first established reliability in using the coding system on two transcripts, reconciling any discrepancies, then independently coded the remaining transcripts. Each speaking turn was treated as a separate quote, and each quote was given 0–13 codes. Transcripts were combined and sorted by code. The transcripts, quotations, and codes were managed using Microsoft Excel 2016 and SPSS version 27.0.

## 3. Results

### 3.1. Patients

Mean patient age at interview was 72.4 ± 6.3 years (64.9–85.0) and mean PD medication and illness duration was 14.4 ± 1.9 years (11.7–17.1; Table 1). All patients were male and Caucasian. The cohort included 10 patients from the early DBS trial: 4 randomized to early-DBS+ODT (eDBS) and 6 randomized to early-ODT (3 were medically treated (eODT); 3 received DBS as standard care after the trial concluded (eODTsDBS)). Quality of life (PDQ-39) at baseline was 15.2 ± 6.0 and increased to 25.9 ± 13.3 at the 11-year visit (Table 1).

### 3.2. Framework Overview

**Figure 2:**
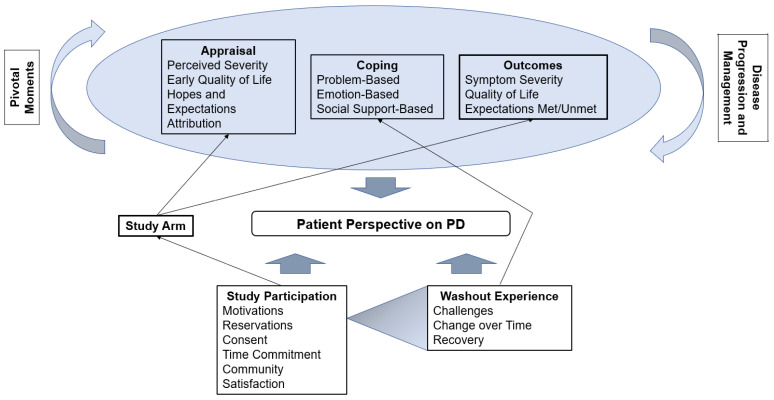
**Conceptual framework**. The early DBS trial participants’ perspective on their PD is determined by a cycle of appraisal, coping, and outcomes. This cycle is influenced by their participation in the trial (study arm) as well as disease progression and management as well as pivotal moments of living with PD.

This study followed an iterative inductive/deductive approach to qualitative research [18,19]. Inductively sorted, coded quotes were used to identify themes and relationships between themes specific to this study. A conceptual framework was developed from this analysis (Figure 2). Deductively, coding the patients’ experiences was approached using a biopsychosocial framework [20,21] social cognitive theory [22,23], and a psychological model of stress and coping [24,25,26].

Figure 2 presents a conceptual framework we developed as a part of our qualitative analysis to understand a patient’s perspective on his PD: patients experience cycles of appraising their condition, coping with their new reality, and experiencing outcomes. Upon initial PD diagnosis, patients appraise their current symptom severity and quality of life and formulate hopes and expectations for the future. They develop problem-, emotion-, and social support-based coping strategies to deal with PD-related life changes, and, as their disease progresses, assess their new symptom severity and quality of life, reflecting on whether their expectations were met. Over many years of living with PD, this cycle is driven through additional revolutions by pivotal moments, disease progression, and treatment decisions. This cycle shapes a patient’s perspective on his PD. In this unique patient population, study participation (including randomization assignment/study arm) and the washout experience also contributed to PD patient perspective, both by interfacing with the cycle of appraisal, coping, and outcomes as well as by separately inducing reflections on the PD experience.

### 3.3. Study Participations

#### 3.3.1. Motivations

A key motivation for enrollment was potential benefit of early DBS. Desire to maintain careers or independence and prevent disability contributed to this motivation. Some patients discussed hopes of slowing disease progression:

My hope was that [DBS] would slow or stop progress of the disease. At first, when I was diagnosed, [my] neurologist said, “Well, you probably ought to start thinking about getting your affairs in ordering and retiring,” which I didn’t like the idea of that. So, anything that would slow the progress of disease, I was all for. (Participant 9, eDBS)

Some described early DBS as an additional tool to combat PD:

Knowing what DBS was intended to do, it just concerned me that it was not made available to people who were diagnosed with Parkinson’s and otherwise healthy, and if it could be an extra tool to help combat the disease or make things easier, then why not make it available to people with early [stage PD]. I was quite hopeful during this selection process that I would get selected and [was] glad I did. (Participant 7, eDBS)

Motivation for others stemmed from a desire to help future PD patients:

I regarded it as an altruistic move on my part, to take part in the study. I did not see... how I was going to benefit from it at all… But it would provide useful information for the curing of Parkinson’s. (Participant 1, eDBS)

#### 3.3.2. Reservations

Hesitance to accept the risks of brain surgery while still functioning adequately with PD emerged as reservation about study enrollment:

He was functioning just fine, at that time. He continued to drive, he continued to do his activities of daily living independently... one of the doctors said that they had expected that they wouldn’t have enough volunteers because who would want to have brain surgery when they weren’t having any problems? Because that can be a scary process, regardless of your diagnosis. (Participant 1, eDBS, Caregiver)

The surgery itself caused hesitation for some but not for all:

The thought of having brain surgery while still conscious was not very appealing to me. (Participant 1, eDBS)

I didn’t hesitate to get the operation. … I was not afraid of it... I think some people would be bothered by the idea of brain surgery, but it didn’t bother me. (Participant 9, eDBS)

Uncertainty surrounding PD also caused hesitation:

Well, with my wife, there were concerns in the family as to whether or not I was doing the right thing. Again, back then, not a lot known about Parkinson’s. (Participant 7, eDBS)

#### 3.3.3. Informed Consent

Patients discussed the thoughtful nature of the expanded, three-part informed consent process led by a medical ethicist [17]:

It’s not something that once you sign a piece of paper, they say, “Well, come on. We’ll do it.” With the study, it’s a very thoughtful process. (Participant 7, eDBS)

One patient expressed determination to receive DBS that did not waver during the consent process:

Of course, once I understood the program, I was very anxious to get the device, be proactive about it... I remember saying [to the medical ethicist], “I understand that there’s a 5% chance of a brain bleed during the operation, but I also understand there’s a 30% chance of early-stage dementia with Parkinson’s. What would you do? It’s an easy decision for me.” (Participant 9, eDBS)

#### 3.3.4. Time Commitment

When considering whether they would recommend participation in a similar study to others with PD, a caregiver noted the significant time, emotional commitment, and perseverance required:

If I told them to consider it, I hope they would be in it for the long haul, because trust me, five years is a long time going every six months. If they’re going to go, I would hope that they would stick it out and finish it, because that’s the only way that, I guess, we got this far. I mean, we’ve stuck it out not only spiritually, but mentally... we didn’t miss a time. And yes, it was stressful, because we knew six months from that time, we would be going back into the hospital. (Participant 10, eODTsDBS, Caregiver)

For one, the week-long washouts and recovery periods interfered with work:

I would not feel what I considered back to where I had been prior to the stay for maybe a week. And that’s really what caused me to step back from my position at work at that time. Because I was out eight days and it’d take me about a week to get straightened out. So every six months, I was two weeks out. And when you’re on production quotas and recruiting quotas and you get so far behind, then it’s just a stressful situation trying to keep up. (Participant 3, eODTsDBS)

#### 3.3.5. Community

Camaraderie and shared experience emerged as prominent and enjoyable aspects of study participation:

It was really, to be honest, the first time or the second time I was there, I found it very comforting to have other guys around with similar problems. (Participant 10, eODTsDBS)

My wife and my family were very sympathetic, but they couldn’t really understand what I was going through. These guys could. So going in and staying for eight days at a time off medication was a challenge. But it was interesting to have other folks there to do that with… Yeah, that’s been a lifelong impact. … I expect to be connected to those guys. As long as you struggle with this thing. (Participant 3, eODTsDBS)

Patients expressed a sense of connection to the PD community and its resources:

The best thing that happened to me recently was meeting [study neurologist] and getting into the program. That gives me people to associate with who are positive and enthusiastic and it gives me some directions. It’s a shame that everyone who has Parkinson’s can’t be connected with the people that have the best advice like I have. (Participant 6, eDBS)

#### 3.3.6. Satisfaction

Despite challenges with the washout periods (see Washout Experience), patients largely expressed satisfaction with trial participation. Satisfaction was discussed in terms of feeling taken care of, potential benefit to others, and empowerment:

He was taken very good care of and he always feels very safe and that eventually, somehow, this is going to help. (Participant 4, eODT, Caregiver)

It’s just been neat to have opportunities to feel like you have a framework in which to fight back against this disease. (Participant 3, eODTsDBS)

An early DBS patient noted subsidization of surgery costs as part of being in the clinical trial:

I would say if that’s the only way you can get the device during the early stages, I’d say do it for sure… I would recommend that people get deep brain stimulation early, by hook or by crook. I realize that … I got [the] operation at least, for free, so I certainly appreciate that. That was worth the investment of my time. (Participant 9, eDBS)

Randomization into the control group was regarded as a disappointment:

There were some very disappointed patients... some were very upset that their number was not chosen as one of the surgery patients. (Participant 1, eDBS, Caregiver)

I did not receive it [DBS], originally. … It would have been nice if I’d had it sooner, but in reflection, I guess it’s kind of worked out just about right. They advanced… the electrodes going into the skull... I got one of the later models. (Participant 10, eODTsDBS)

### 3.4. Washout Experience

The washout involved stopping the use of PD medications and, if applicable, turning off DBS devices for seven days to assess physical and cognitive symptoms of PD in the absence of treatment. Washouts were completed at baseline and every six months during the pilot trial (early-stage PD) and attempted again at the eleven-year study visit (mid/late-stage PD; Figure 1). Patients were asked about their experiences with the washout protocol at both stages of their disease.

See Table 2 for patient quotations.

#### 3.4.1. Washouts: Early-Stage PD, Years 0–2

Every week-long washout was completed during the two-year trial [14]. For most, the early-stage PD washouts were characterized by the gradual worsening of PD-specific symptoms, such as tremor, stiffness, and bradykinesia, throughout the week (Table 2). One patient found the washout emotionally difficult, while for others stopping treatment had no significant effect or even alleviated certain symptoms.

Coping strategies during the early washouts included social activities among patients and physical activity. Readjustment to treatment after the washout occurred without significant issues, and patients typically felt back to normal in a week or less.

#### 3.4.2. Washouts: Mid/Late-Stage PD, Year 11

Patients described the later-stage PD washout as significantly more challenging (Table 2). Six of nine patients interviewed chose to end the washout before reaching the full 7 days (mean washout duration: 5.2 days; Table 1). Many patients experienced significant exacerbation of physical symptoms and reduced ability to independently perform activities of daily living. Multiple patients also found the later-stage washout emotionally difficult, and others also experienced physical challenges that made it difficult for them to complete study assessments.

Patients emphasized the importance of support from the CRC staff in making the later-stage washout safe and feasible. Newfound recognition of how difficult life with PD would be without DBS and/or medication emerged as a primary theme during reflection.

### 3.5. Appraisal, Coping, and Outcomes

Table 3 presents themes of appraisal, coping and outcomes that emerged across all PD patients (Figure 2). Experiences unique for early DBS patients are described below.

#### 3.5.1. Appraisal

Patients recalled their appraisal of their condition in the period following diagnosis in terms of perceived severity, hopes and expectations, early quality of life changes, and attribution of their symptoms to PD compared to normal aging (Table 3).

##### Early DBS

Patients randomized to receive early DBS also recalled hopes and expectations specific to this treatment:

I hoped that [DBS] would … slow down my Parkinson’s, and make me be able to maneuver around without the albatross of having wires hanging over my head. (Participant 6, eDBS)

[I hoped] that I would not become more dependent on others, that I would still be capable of dealing so much. (Participant 7, eDBS)

I did not know... how I dreamt of it. I just assumed it [DBS] would do no harm. (Participant 1, eDBS)

#### 3.5.2. Coping

Common coping strategies for living with PD included information seeking, establishing a medication routine to make dosage-related symptomatic variability more predictable, and finding tricks to manage individual symptoms (Table 3). Social support and emotion-focused strategies, such as acceptance, faith, social comparison, perseverance, and positivity, were used to cope with the psychological impact of progressive illness.

##### Early DBS

Early DBS patients utilized similar coping strategies, as well as additional methods particular to their treatment. While they employed similar medication management techniques as those randomized to ODT, some expressed relief at the constancy of DBS:

The problem I have with [medications] is remembering to take them and trying to remember if I did take a dose and remembering to take the next dose. Fortunately, with the DBS, once it’s on, it’s on. (Participant 7, eDBS)

Adjusting DBS settings provided new opportunities to control symptoms, though this also required a learning period for patients:

Our goal when we went to [doctor], was to get him to enable [patient] to speak more clearly. That was my goal that I stated. That if he could just speak more clearly and they did indeed see that an adjustment could be made for that purpose. And he does speak more clearly now. (Participant 1, eDBS, Caregiver).

At one point, the settings might have been a little bit too high. So it just seemed to intensify my movements. Dyskinesia… I believe at one point, they decided to move from one set of leads down to another in their fine-tuning. I think that helped a lot. Again, it’s a learning process for the patient and the doctors. (Participant 7, eDBS).

#### 3.5.3. Outcomes

##### Symptom Severity and Quality of Life

As PD progressed over the 11-year period, patients experienced a range of symptom severity and quality of life outcomes (Table 3). Those who received DBS as part of their standard of care treatment noticed clear symptomatic benefits of the treatment.

##### Early DBS

Similar to standard of care DBS patients, one early DBS patient noticed DBS-related alleviation of specific symptoms, such as tremor and stiffness:

Well, it seemed to help a lot with my tremors in the hand and the tenseness, stiffness in my hands and feet and shoulders. That was one thing that just went away. That was no longer a huge event. So that was the biggest thing. (Participant 7, eDBS)

More often, however, early DBS patients described minimal noticeable impact in the early months of their DBS:

He didn’t feel different than when he didn’t have it, other than his tremor was ended. (Participant 1, eDBS, Caregiver)

Well, I remember going to [the study neurologist] … after surgery and feeling that I was disappointed that I could see no improvements in 30 days... I’m only saying that because the light bulb just went on. I totally understand now. I get it, what’s going on. I went in one day and said, “I understand how my brain is working to support the DBS.”... I expected it to be like turning it on and everything will be super. Instead, DBS, it didn’t work. It was a step forward success. It wasn’t a great success. It was another tool that we used to move forward. (Participant 6, eDBS).

Instead, the impact of early DBS was discussed in terms of prevention of major quality of life changes due to PD:

Since I had the DBS, everything’s going kind of smoothly. I’m anticipating it getting worse, and quite frankly, I’m at a point it’s starting to slow me down and I’m feeling the effects of Parkinson’s. But up until recently, I was really unaware every day that I had Parkinson’s… It helped me in my attitude. It gave me a lot of confidence because I knew that I had an extra gun in my pocket. (Participant 6, eDBS)

So far, [PD has] not affected me very much. I still work full time as a lawyer. I still play golf, a lot. It’s not affected much about my everyday life. It’s a little more difficult to put on my pants, for example, because of balance problems, but in general, have not been affected very much. (Participant 9, eDBS)

The inability to identify specific ways DBS was helping emerged as a theme among early DBS patients:

It still prevents that tremor in the right hand. I don’t know that it’s helping me in any other way, because it’s hard to say. Because if it is, it’s been doing it for a long time and it’s not something that I notice any longer. (Participant 1, eDBS)

I have always felt it probably mitigated some symptoms that he might have if he did not have the DBS, but again that’s nebulous because we don’t know what those would be. We never turn [the DBS] off. (Participant 1, eDBS, Caregiver)

However, the washout experience revealed symptoms that were normally masked by DBS and medication:

When I was off the device… I could tell that my coordination was substantially compromised. But with the medication and on the device, I didn’t have any particular trouble whatsoever, and most people would not know that I have Parkinson’s disease. (Participant 9, eDBS)

Balance, handwriting, speech, constipation, drooling, freezing, and cognition were symptoms that early DBS patients wished their DBS would treat better.

##### Expectations Met/Unmet

Standard of care DBS patients expressed recognition that DBS was not a cure-all and felt satisfied with their treatment despite continuing to struggle with symptoms (Table 3).

##### Early DBS

Similarly, all early DBS patients (4/4) interviewed expressed satisfaction with receiving early DBS and felt comfortable with the timing of the treatment:

It was the best thing that I had ever done. A very positive thing for me. (Participant 6, eDBS)

Glad I started early on, would not want to have done it any other way. I know it’s not a cure-all, but I’m glad I got it. To have waited till I got really bad with no hope that there would be no turning back any progression. I would not have liked being put in that situation… I don’t see any negativity with having DBS in my sense at an early stage. (Participant 7, eDBS)

It was early in the diagnosis, but it was not early in my age, I was 74 years old and—I believe that was okay… I appreciate it today, the effect from that. (Participant 1, eDBS)

Multiple patients felt satisfied because they believed that early DBS slowed their disease progression:

Medication that’s available now has not been shown, I believe in any way to slow or stop progression. That’s why the study was done for early [DBS] to see what impact it does possibly have on slowing or stopping progression. In my case, I would have to say it does. But that’s me. In my unique Parkinson’s situation, I believe it has slowed. (Participant 7, eDBS)

It’s been 16 years [since joining the study] and until recently, you literally couldn’t tell I had Parkinson’s... I told [study neurologist] the other day … “I probably wouldn’t be here today,” if I hadn’t had all this exchange with him and the team, I probably would have passed away, I think. (Participant 6, eDBS)

All early DBS patients (4/4) said they would recommend early DBS treatment to others:

I’d tell them to not hesitate, to do [early DBS] for sure. (Participant 11, eDBS)

DBS I see not having any kind of a negative factor in any of those situations. I would encourage it greatly… I would encourage anyone that had a noticeable impact in their life due to Parkinson’s, a confirmed diagnosis regardless of whether or not medication was tolerable or they thought it was making a good impact… knowing that medication is time-based, it’s not constant, and Parkinson’s is a progressive disease. (Participant 7, eDBS)

### 3.6. PDQ-39 Quality of Life Assessment

Table 1 presents PDQ-39 scores at baseline and 11 years. Quality of life assessment scores worsened for most participants over the 11-year study period (n = 2 eDBS; n = 3 eODT; n = 1 eODTsDBS). Only participants with DBS (n = 1 eDBS; n = 2 eODTsDBS) had PDQ-39 scores improve over 11 years.

## 4. Discussion

This is the first qualitative study evaluating patients who received DBS in very early-stage Parkinson’s disease. In addition to patients who received early DBS, patients randomized to optimal drug therapy also reflected on their experience participating in the DBS in early-stage PD clinical trial. This study not only provides insight into the patient experience of early DBS over 11 years but is also the only qualitative study to examine the long-term experience with DBS alongside patients randomized to a medication control group.

Two prevailing motivations for enrollment in the early DBS trial arose in this study: (1) desire to help future PD patients by participating in research and (2) potential benefits of early DBS (i.e., maintaining careers and independence, preventing disability, and slowing disease progression). Prior studies investigating whether standard-of-care DBS patients would have preferred earlier DBS similarly cited slowing progression and regaining freedoms sooner as motivators, with the additional motivation of eliminating medication side effects earlier [27]. In contrast, key motivators for patients considering standard-of-care DBS include their worsening symptoms, family encouragement, and confidence in the recommendation of the physician [28,29].

Major hesitations for enrollment in the early DBS trial related to surgery: risk of peri-operative adverse events and receiving surgery while awake. Fear of adverse events is also cited as the major concern for those considering standard-of-care DBS, although the extent of this fear differs among patients [28,29]. This concern is likely to carry even greater weight in a population considering early DBS, as patients are still functioning well and do not yet see DBS as a last resort. While some PD patients certainly express hesitation to consider early DBS [27], there is a notable proportion of patients who indicate that the potential benefits may outweigh the risks [27,29,30,31]. Currently, early DBS is appropriately only offered in research studies, and a pivotal safety and efficacy study is needed to understand if DBS should be offered earlier in Parkinson’s disease.

Patients in this study noted the enhanced informed consent process made them feel educated about participating in the clinical trial [17], which may have contributed to the sense of commitment mentioned by some patients. Notably, there was a remarkably low drop-out rate in the early DBS trial through five years of follow-up (3.3%, 1/30). Comradery was also noted in this study, which, in addition to being well-educated during the consent process, may also have contributed to the low dropout rate in the two-year trial.

### 4.1. Washout Experience

The use of repeated, week-long therapeutic washouts was a trial design feature that permitted evaluation of the underlying symptom progression of PD and ultimately led to Class II evidence that early DBS slows the progression of rest tremor [32]. All patients who finished the two-year trial completed all five week-long washouts. This study reveals that while the early-stage washouts involved challenges due to time commitment and symptom exacerbation, the mid-/advanced-stage washouts were described by patients as significantly more difficult both physically and emotionally. Two-thirds of the patients interviewed in this study (6/9) elected to end the 11-year washout early. As expected, due to milder symptoms, early-stage PD washouts are much better tolerated, and therefore more feasible to implement in clinical trials.

### 4.2. Early DBS and Standard of Care DBS

Patients randomized to early ODT in the trial but who later received standard-of-care DBS articulated significant improvement in symptoms following DBS implementation. This is consistent with prior reports, where the post-DBS period for standard-of-care patients is often characterized by dramatic improvement in symptoms and quality of life that patients describe as “miraculous,” “feeling reborn,” or a “new chapter in life” [9,33,34]. Symptoms become milder and more predictable, enabling patients to participate in activities they had not previously been able to manage [33,34].

In contrast, early DBS patients did not describe immediate, impactful improvement to specific symptoms, aside from one patient whose tremor was eliminated. Instead, early DBS patients spoke of the stability of their lives despite living with PD, commenting that for many years after DBS, they were not greatly affected by their PD or were even “unaware” on a daily basis that they had PD. Unlike the standard-of-care DBS patients in this study as well as in prior reports [9,33,34], early DBS patients were often unable to identify specific ways that DBS helped them, instead speculating that DBS prevents symptoms that they would have experienced without the treatment. DBS is currently applied during mid- and advanced-stage PD, a time in which medications have become less effective and patients are experiencing symptoms that severely impact their quality of life [9,29]. Given that early DBS patients began DBS before reaching this stage, it is unsurprising that they did not experience as drastic a change in symptoms and quality of life due to DBS as did their standard-of-care counterparts. Instead, early DBS may lead to a maintenance of quality of life that is less noticeable after surgery.

More than ten years after receiving the therapy, all early DBS patients expressed satisfaction with receiving DBS, felt comfortable with its timing, and would recommend it to others. This mirrors high satisfaction rates seen with standard-of-care DBS patients [27,35,36], although satisfaction may depend on adjusting expectations [35] and may decline moderately over time with declining treatment efficacy [27]. A greater sample size and intermittent surveys of satisfaction are necessary to see if these effects carry over to early DBS patients.

### 4.3. Qualitative and Quantitative Analysis of Quality of Life

Nearly all participants interviewed (9/10) also completed the PDQ-39 at baseline and 11-years. Interview responses related to quality of life for many participants echoed the 11-year change in PDQ-39, with qualitative descriptions offering a complimentary perspective on factors influencing quality of life beyond the single PDQ-39 summary index score. Three participants had PDQ-39 scores at the 11-year visit that were better than when they enrolled in the trial as very early-stage PD patients. All three had received DBS (1 eDBS, 2 eODTsDBS), and this finding is consistent with quality of life improvements after DBS when it is applied in more advanced-stage PD patients [6]. Notably, the participant with the greatest worsening in PDQ-39 score (Participant 7, eDBS) reported high satisfaction with early DBS, even remarking on how he believes it has slowed progression of his Parkinson’s disease. Combining the validated PDQ-39 quality of life assessment data with the rich descriptions offered in this study offers the opportunity to discover important domains of living with Parkinson’s that are not adequately captured using the PDQ-39 alone. Integrating qualitative measures alongside quantitative assessments provides added perspective in the understanding of quality of life.

### 4.4. Limitations

Results from this study should be interpreted with caution due to its small sample size of only ten patients (one-third of original trial participants). Experiences of patients from the pilot trial who were not interviewed, either due to loss to follow up (n = 3), disability (n = 2), death (n = 12), inability to coordinate with caregiver (n = 1), or declination (n = 1), may differ from that of those in this study. Although the sample size is too small for generalization, describing these patient experiences is important, because this is the only existing cohort that has received very early-stage DBS therapy. Results from this small study are hypothesis-generating, and additional research in a larger cohort of PD patients is needed to confirm the importance of the relationships presented in our conceptual framework. Finally, recall bias should be considered since interviews asked patients to remember over an 11-year time frame.

## 5. Conclusions

This study describes the patient experience of participating in the only clinical trial of DBS in early-stage PD conducted to date. Participants were motivated to enroll in the pilot trial by desire to help future PD patients and the potential to benefit from early DBS therapy, although some hesitated due to the risk of surgical adverse events. Patients expressed satisfaction with their participation, and those who received early DBS were satisfied with their treatment. This study suggests that the experience of early DBS differs substantially from that of standard-of-care DBS due to the difference in the disease experience at the time when the treatment is applied, with early DBS being described as maintaining, rather than drastically altering, quality of life. More investigation is needed to understand the patient experience after receiving early DBS, and the FDA has approved a prospective, randomized, double-blind, pivotal clinical trial evaluating DBS in early-stage PD (IDEG050016).

## Figures and Tables

**Table 1:** Patient information.

		At Enrollment			At 11-Year Interview				
Patient	Treatment Group *	Medication Duration (Years)	Age (Years)	PDQ-39 Summary Index	DBS Duration (Years)	Medication Duration (Years)	Age (Years)	PDQ-39 Summary Index	11-Year Washout Duration (Days)
1	eDBS	1.0	73.9	7.5	10.4	12.1	85.0	d.n.p.	d.n.p.
2	eODTsDBS	0.9	63.2	19.5	5.2	14.3	76.6	15.2	4
3	eODTsDBS	3.9	55.5	14.6	0.5	16.8	68.3	14.3	5
4	eODT	3.0	59.7	9.6	n.a.	15.6	72.3	15.6	7
5	eODT	1.3	67.9	24.9	n.a.	12.2	78.8	36.8	w.n.a.
6	eDBS	1.7	56.4	19.7	13.0	15.0	69.7	18.1	w.n.a.
7	eDBS	0.6	55.4	14.3	10.9	11.7	66.5	51.8	7
8	eODT	1.4	52.3	14.5	n.a.	14.1	64.9	30.0	7
9	eDBS	1.4	53.6	6.8	13.3	15.2	67.4	15.8	2.5
10	eODTsDBS	3.5	60.8	20.3	7.6	17.1	74.4	35.2	4
Mean ± SD (Range)		1.9 ± 1.2 (0.6–3.9)	59.9 ± 6.8 (52.3–73.9)	15.2 ± 6.0 (6.8–24.9)	8.7 ± 4.6 (0.5–13.3)	14.4 ± 1.9 (11.7–17.1)	72.4 ± 6.3 (64.9–85.0)	25.9 ± 13.3 (14.3–51.8)	5.2 ± 1.7 (0–7)

* eDBS = randomized to early DBS+ODT; eODT = randomized to early ODT and never received DBS; eODTsDBS = randomized to early-ODT and later received standard of care DBS after the trial concluded; d.n.p. = did not participate in 11-year study; w.n.a. = 11-year washout not attempted.

**Table 2:** Thematic factors and representative quotations significant in the washout experience.

Theme	Representative Quotation
**Washouts: Early-Stage PD** **Years 0–2**	
Gradual symptom worsening	- My left arm definitely shook more, and I started getting very stiff. (Participant 8, eODT) - I never really had a lot of tremor, but I would say that I did develop tremor. Doing the hand dexterity tests, the clapping the fingers together and turning the hand over and back, it got worse and worse. (Participant 9, eDBS) - I just got very slow. And in the last couple of days I could get out of bed, but I didn’t want to. (Participant 3, eODTsDBS)
Emotional difficultly	Depression was the biggest thing for me. Huge depression. (Participant 6, eDBS)
No significant effect/alleviation of symptoms	- At the time I was not losing anything by it. Stopping the medication didn’t make a difference. The only effect I remember is that I would have trouble sometimes at night sleeping and I would need Benadryl. But that was relatively minor. (Participant 1, eDBS) - He seemed a lot better, but [the study neurologist] explained that it’s kind of typical that when they withdraw the medication that it seems like his symptoms are relieved and he can do things better. (Participant 4, eODT, Caregiver)
Coping Strategies	- We’d still get out and play golf and we would go to a nine-hole course that we could walk. So we didn’t even rent a cart. But as the days would go on, you get pretty stiff and pretty clumsy feeling. (Participant 3, eODTsDBS) - I would go up six flights of stairs and start walking through all the hallways in the building and then go to the next level and do it again and do it again. Just wandering around randomly, getting down to the tunnels and walking… I just try to keep moving, and that helps with the stiffness and the fatigue. (Participant 7, eDBS)
Treatment readjustment after washout	- What I remember is when the device was turned on, I got buzzed through my body… I don’t remember ever having a long period of time before I got to be feeling better, but it was noticeable. (Participant 9, eDBS) - It would take me upwards of a week afterwards to really get feeling adjusted on the medication again. (Participant 3, eODTsDBS)
**Washouts: Mid/Late-Stage PD Year 11**	
Exacerbation of physical symptoms	- This exhaustion. I was so tired after a day of testing that I just didn’t see how I could go on. (Participant 5, eODT) - I couldn’t eat, because I would sling my food all over the place. (Participant 10, eODTsDBS) - I could not do anything for myself. Even get up to urinate. The nurse would have to come in and actually handle everything for me. I just couldn’t do it. And when I would go into the test, I really couldn’t much complete, to any degree, any of the testing. So I just felt like I can’t get much worse. I can’t do anything now. (Participant 3, eODTsDBS)
Emotional difficulties	- The last part of the time, I was sort of in a trance. I knew what was going on around me, but I couldn’t effectively communicate. Again, the [research team was] great, but there’s a limit to how much they can do. It was very difficult going cold turkey off the DBS and all the medications. I was shaking violently at times. Sometimes I would cry... when you start to cry... that’s a very difficult situation to be in. (Participant 10, eODTsDBS) - I remember the last night the nurses would come in. I would ring the bell, because I’d need to go to the bathroom or something. They would come in and they’d ask me a question. I could not respond quick enough for them to even consider that I was able to answer. So it was pretty tough. (Participant 3, eODTsDBS) - I had to leave early. I really got frustrated... I couldn’t even make it to the bathroom and I peed my pants in the middle of the floor. That was very embarrassing and very frustrating and I told [study neurologists] I had to leave. (Participant 2, eODTsDBS)
Difficulty completing study assessments	- I guess the most challenging part was I tried to be consistent in my actions and everything, but as far as being I guess in the test, the Parkinson’s test, I just felt totally clumsy. (Participant 8, eODT) - [The most challenging part was the] brain scan. Trying to stay still for that was problematic. (Participant 7, eDBS)
Importance of CRC staff support	- All [of the] stays, the staff is always available to help us with any issues and knowing that that was there, that I wasn’t going to have to figure out how to do things by myself or if I was not able to get dressed completely or get to wherever food was being served, knowing that that help was always available surely made it a lot easier… Without that kind of support, I don’t know if a study like this would be feasible. (Participant 7, eDBS) - Falling was my biggest problem. I almost fell in the shower. I almost fell out of bed one time. I think that’s why, it wasn’t safe for me to get up by myself. I had somebody in there. I had to have a cane with me at all times. (Participant 10, eODTsDBS)
Newfound recognition of difficulties without PD treatment	- I felt after the drugs were off and the device was off… Parkinson’s would, to me, be pretty debilitating because as I say, I was using a walker to get around the hospital room, and I had trouble turning over in bed. I didn’t like that at all, of course, so I’m thankful to have the device and the drugs, for sure. (Participant 9, eDBS) - I had no idea I had progressed so much from the previous study stays... And so it was pretty eye-opening that, if it were not for the medication and somehow the DBS, I would be... well, I probably wouldn’t be here. But certainly if I was, I’d be bed ridden. So it was pretty eye-opening. (Participant 3, eODTsDBS)

**Table 3:** Thematic factors and representative quotations significant in eleven-year PD experience.

Theme Subtheme	Thematic Description	Representative Quotation
**APPRAISAL—All Patients**		
Perceived Severity	Reaction to diagnosis	- I was pretty overwhelmed. I went home and Googled it. Then all the aspects and ramifications of it looked pretty serious. (Participant 3, eODTsDBS) - It’s not real. It’s not as bad as it sounds… Everything can be fixed. We’re going to fix this. We’ll fix it now. (Participant 4, eODT)
	Perceived severity changed with progression	- Early on it wasn’t so bad, but by about five years, maybe a little later it dawned on me that this was really going to be world-changing. (Participant 5, eODT)
Hopes and Expectations	Expectation of progression	- I had the feeling that it was kind of like we weren’t… looking ahead that it was going to get better, being progressive. To me that’s the hardest. (Participant 4, eODT, Caregiver)
	Desire not to be pitied	- I think getting the word out to my friends and business associates that I had Parkinson’s but was not I mean, to be pitied was really a big thing to me. I didn’t want people to walk around and say, ‘There’s the guy that has Parkinson’s.’ (Participant 6, eDBS)
Early QoL	PD caused embarrassment at work	- I started falling asleep a lot. I remember sitting in a meeting with a business alliance and my boss came to me after and said, “You slept during the meeting.” I said, “Impossible.” I remember everybody talking. But I had fallen asleep. I found the sleepiness and everything to be a challenge for me. (Participant 6, eDBS) - Even still today when I’m going out and eating, my feet and body moves a lot. So at work, it was just kind of, whoever was sitting on either side of me knew they were going to get hit or kicked, or I was going to bump into them, and it was almost a comic event. (Participant 7, eDBS)
Attribution	Patients unsure which symptoms to attribute to PD versus normal aging	- I noticed memory and cognition [are worse], but keep in mind as my neurologist says that can’t blame everything on Parkinson’s. A lot of the other things one might be going through is just typical old age disease, things that occur or start happening as the body gets older. (Participant 7, eDBS)
**COPING—All Patients**		
Problem-Based	Medication management	- [I tried] to establish a routine where I would be able to figure out when to take the medication to hopefully prevent [wearing] off but also trying to place myself in a situation so that when I did take the medication during the day time I would be able to handle any nausea that came up. (Participant 7, eDBS)
	DBS management	- They didn’t tell me how much trouble it would be to change the battery out of [the DBS unit]… We jumped through hoops, because the battery went down on my original one. They ended up replacing my whole unit. Not just putting batteries in it, but they put a whole new unit in. (Participant 10, eODTsDBS)
	Information seeking	- I just felt like I needed to focus on doing the things I could do to have a better outcome, instead of being victimized by it. I did several things. I looked for the research studies that I could fit into that I could have some impact. And that was a huge thing. (Participant 3, eODTsDBS) - Just becoming more aware of how the disease progresses has helped a bit. (Participant 7, eDBS)
	Symptom-specific coping strategies	- If you’ve got lock up, that’s the problem. You start shaking and that’s when you lose your balance. You’ve got to do what [study neurologist] said, you’ve got to step over the big log every time you’ve got to take off to go walking. (Participant 2, eODTsDBS) - I will say, interestingly, that at times, my face loses affect. It’s not an all-time thing, but it’s a sometimes thing, and that’s bothersome. I’ve actually grown a beard as a way to try and cope with that. (Participant 11, eDBS)
Emotion-Based	Acceptance	- All I have to say, I’m not depressed or upset about it, it’s just, I guess it’s what it is. (Participant 2, eODTsDBS)
	Faith	- I’m a person of faith, and I pretty quickly put that you leave it in God’s hands. (Participant 3, eODTsDBS)
	Social comparison	- I had done, I guess, so well, compared to probably many people’s experience. I’d always felt pretty blessed that I had an easier experience with it than some. (Participant 3, eODTsDBS)
	Perseverance/determination	- We hope it goes away, but we know in the back of our mind, it’s not going to go away. But we push ourselves and fight. We’re not like laying down and just letting it come on us for whatever reason. We’re fighting this. We are pushing ourselves to the very end. (Participant 10, eODTsDBS, Caregiver) - In the first five years, even to learn all about Parkinson’s was... you didn’t get mad. There were going to be a lot of good days and tuck the bad things away and leave them for another day. (Participant 6, eDBS)
	Positivity	- What I’ve done is I’m trying to maintain a positive outlook all the time. I’m the most positive guy as far as this is concerned of anyone around. I don’t want to be known as the guy who has Parkinson’s. I think my good, positive attitude has really made a positive result for me throughout the whole process. (Participant 6, eDBS)
Social Support-Based	Caregiver impact	- I have a very good caregiver here... It makes a big difference in my life. (Participant 1, eDBS)
	PD support groups	- There’s a Parkinson’s support group here in [city] that until the COVID thing, we had breakfast twice a month. That was interesting and helpful, and I’ve developed some good friendships in that way. (Participant 9, eDBS)
**OUTCOMES—Standard of Care DBS Patients**		
Symptom Severity	Noticed clear symptomatic benefit after receiving DBS	- It helps in the walking, helped me with the tremors, and the locking up. It helps with the freezing up and all of that. It’s a benefit to everything, it’s good. (Participant 2, eODTsDBS) - You don’t fall. Like, you fall, but not like... not every time you get up, you don’t fall. You can still do your shower and you don’t fall in the tub. (Participant 10, eODTsDBS)
	Accepted that DBS is not a cure-all	- It would have been nice if it would have treated the cognitive issues that I have... if it had more effect on those. Again, I went into it knowing what DBS would do and what it wouldn’t do, so I didn’t have any preconceived ideas about that at all. (Participant 10, eODTsDBS)
Quality of Life	Drastically improved quality of life in the short-term	- At that time, it was kind of like, man! We’ve got this kicked… We got to do a lot of things that we normally probably wouldn’t have ever done. We got to go places, and see things, and it was the best thing ever... I mean, I guess the truth actually hits us after a little while and then, we’re just kind of taking it step-by-step now. (Participant 10, eODTsDBS)
Expectations met/unmet	Satisfied with DBS treatment despite continued symptoms	- Everything about my whole life is, particularly mentally and I guess emotionally and psychologically it’s all affected. But I couldn’t say enough good things about the deep brain stimulation surgery. And the fact is you may, you know, even with all that I’m dealing with it’s much better than it would have been if I hadn’t gotten it. (Participant 2, eODTsDBS)

## Data Availability

Not applicable.
